# Intact glycopeptides identified by LC-MS/MS as biomarkers for response to chemotherapy of locally advanced cervical cancer

**DOI:** 10.3389/fonc.2023.1149599

**Published:** 2023-07-13

**Authors:** Jing Li, Xiaoxiao Feng, Chongying Zhu, Yahui Jiang, Hua Liu, Weiwei Feng, Haojie Lu

**Affiliations:** ^1^Department of Obstetrics and Gynecology, Ruijin Hospital, School of Medicine, Shanghai Jiaotong University, Shanghai, China; ^2^Department of Chemistry and NHC Key Laboratory of Glycoconjugates Research, Institutes of Biomedical Sciences, Fudan University, Shanghai, China; ^3^Department of Laboratory of Obstetrics and Gynecology, Ruijin Hospital, School of Medicine, Shanghai Jiaotong University, Shanghai, China

**Keywords:** neoadjuvant chemotherapy (NACT), locally advanced cervical cancer (LACC), glycopeptides, LC-MS/MS, biomarker

## Abstract

**Objective:**

For locally advanced cervical cancer (LACC), patients who respond to chemotherapy have a potential survival advantage compared to nonresponsive patients. Thus, it is necessary to explore specific biological markers for the efficacy of chemotherapy, which is beneficial to personalized treatment.

**Methods:**

In the present study, we performed a comprehensive screening of site-specific N-glycopeptides in serum glycoproteins to identify glycopeptide markers for predicting the efficacy of chemotherapy, which is beneficial to personalized treatment. In total, 20 serum samples before and after neoadjuvant chemotherapy (NACT) from 10 LACC patients (NACT response, n=6) and NACT nonresponse, n=4) cases) were analyzed using LC-MS/MS, and 20 sets of mass spectrometry (MS) data were collected using liquid chromatography coupled with high-energy collisional dissociation tandem MS (LC-HCD-MS/MS) for quantitative analysis on the novel software platform, Byos. We also identified differential glycopeptides before and after chemotherapy in chemo-sensitive and chemo-resistant patients.

**Results:**

In the present study, a total of 148 glycoproteins, 496 glycosylation sites and 2279 complete glycopeptides were identified in serum samples of LACC patients. Before and after chemotherapy, there were 13 differentially expressed glycoproteins, 654 differentially expressed glycopeptides and 93 differentially expressed glycosites in the NACT responsive group, whereas there were 18 differentially expressed glycoproteins, 569 differentially expressed glycopeptides and 99 differentially expressed glycosites in the NACT nonresponsive group. After quantitative analysis, 6 of 570 glycopeptides were identified as biomarkers for predicting the sensitivity of neoadjuvant chemotherapy in LACC. The corresponding glycopeptides included MASP1, LUM, ATRN, CO8A, CO8B and CO6. The relative abundances of the six glycopeptides, including MASP1, LUM, ATRN, CO8A, CO8B and CO6, were significantly higher in the NACT-responsive group and were significantly decreased after chemotherapy. High levels of these six glycopeptides may indicate that chemotherapy is effective. Thus, these glycopeptides are expected to serve as biomarkers for predicting the efficacy of neoadjuvant chemotherapy in locally advanced cervical cancer.

**Conclusion:**

The present study revealed that the N-glycopeptide of MASP1, LUM, ATRN, CO8A, CO8B and CO6 may be potential biomarkers for predicting the efficacy of chemotherapy for cervical cancer.

## 1 Introduction

Cervical cancer remains the second most common cause of both cancer incidence and mortality in developing countries (1–3). Data from the National Cancer Institute Surveillance, Epidemiology and End Results Program (NCI SEER) reported that cervical cancer with 14100 new cases in 2022 and a 5-year relative survival of 66.7%, and it is very necessary to tailor them the more appropriate therapeutic and surveillance program (4, 5). Currently, concurrent chemoradiation (CCRT) is considered a standard therapy for locally advanced cervical cancer patients. However, as the Cochrane meta-analysis reported, CCRT shows a stage-dependent advantage over radiotherapy with 5-year survival benefits of 10% for women with stage IB to IIA cervical cancer, 7% for women with stage IIB cervical cancer and 3% for women with stage III to IVA cancer. Therefore, there are limitations in using CCRT for LACC treatment (6, 7).

Recently, several phase II trials have revealed that neoadjuvant chemotherapy (NACT) followed by chemoradiation exerts favorable outcomes in LACC. A weekly regimen of NACT followed by CCRT may be superior to CCRT alone (7–9). The overall NACT response rate ranges from 52% to 95% in different studies (10). The usage of NACT before radiotherapy may potentially eradicate subclinical distant metastasis, reduce the tumor size and correct pelvic anatomy distortion, ultimately allowing better delivery of the following therapy.

Combined analysis has shown that a better clinical response and pathologic response to NACT are associated with favorable PFS and OS (11). Stable disease post-NACT has also been identified as a poor prognostic sign (12). If these patients do not benefit from NACT, alternative therapies may be offered at an earlier stage. To improve the quality of life of nonresponders and avoid inherent resistance to chemotherapy/radiotherapy and potential toxicity as well as reduce the time until radiotherapy and cost, it is necessary to identify biomarkers to predict the efficacy of NACT.

Currently, there are no Food and Drug Administration (FDA)-approved biomarkers that can be used to predict the effectiveness of chemotherapy. Glycosylation is the most common posttranslational modification of proteins, and the decoration of the protein with a variety of glycans leads to the diversity of protein structures (13). Recently, advanced technologies have been developed and applied to the analysis of glycosylation of proteins (14), which has led to an understanding of the diversity and differences in glycans in certain proteins, suggesting that site-specific glycosylation of proteins play important roles in physiological and pathological functions (15). Aberrant glycosylation of glycoproteins is one of the most frequent changes that occurs in the cancer biological system (16–19). The importance of this phenomenon is to provide better survival conditions for cancer cell invasion. Changes in glycans include lost or excessive expression of certain structures, incomplete/truncated structures, accumulation of precursors and/or altered glycan expression (20). Modified glycosylation patterns are associated with cancer invasiveness and metastatic potential (21). Most of the FDA-approved biomarkers for cancer diagnosis and monitoring are glycoproteins (22). Because glycoproteins are often present on the cell surface or secreted from cells, they are present in serum and can serve as biomarkers. Therefore, discovering specific glycoproteins as biomarkers for differentiating the effectiveness of chemotherapy in cervical cancer is crucial.

Based on a clinical trial we are conducting (ethical number N-2018-239), we collected pre- and postchemotherapy serum samples from patients who received NACT. We used MS-based glycoproteomics analysis for differential determination of glycosylation composition changes at individual glycosites in whole serum between patients with and without NACT response. This approach enabled the identification of a panel of N-glycopeptides as potential biomarker candidates for chemotherapy efficacy in locally advanced cervical cancer.

## 2 Methods

### 2.1 Materials and reagents

Ammonium bicarbonate (ABC), dithiothreitol (DTT), iodoacetamide (IAA) and trifluoroacetic acid (TFA) were purchased from Sigma-Aldrich (MO, USA). ZIC-HILIC particles and HPLC-grade acetonitrile (ACN) were purchased from Merck (Darmstadt, Germany). Sep-Pak C18 Vac cartridges were purchased from Waters (MA, USA).

Sequencing-grade trypsin was purchased from Hualishi (Beijing, China). Distilled water was purified by a Milli-Q system (Milford, USA). All other chemicals and reagents of the best available grade were purchased from Sigma-Aldrich (MO, USA).

### 2.2 Sample collection

We collected serum samples from patients before and after neoadjuvant chemotherapy. Patients were eligible if they presented with histologically confirmed squamous carcinoma or adenocarcinoma of the cervix stage FIGO IIB to IIIC. Patients were assigned to NACT with weekly cisplatin and paclitaxel followed by CCRT from December 2018 to January 2020. NACT consisted of intravenous paclitaxel (60 mg/m^2^) and cisplatin (40 mg/m^2^) on Days 1, 8, 15 and 22. For American Joint Committee on Cancer staging, pelvic magnetic resonance imaging (MRI) and PET/CT were performed. The chemotherapeutic response was scored at 7-10 days after the last course according to the RECIST v1.1 criteria as follows: complete resolution of the tumor (CR); partial response (PR, >30% decrease in the longest diameter); stable disease (SD, <30% decrease or >20% increase in the longest diameter); and progressive disease (PD). To reduce the effect of measurement error on the study results, imaging data from all patients were measured by the same radiological physician. The study was approved by Shanghai Jiaotong University School of Medicine affiliated with Ruijin Hospital Ethics Committee (N-2018-239) (7). Signed informed consent was obtained from all patients.

### 2.3 Serum samples

Patients were grouped according to chemotherapeutic effectiveness by RECIST v1.1 criteria (23). We collected paired serum samples from 10 patients before and after neoadjuvant chemotherapy with 6 patients classified as NACT response (CR/PR) and 4 patients classified as NACT nonresponse (SD/PD). In the present study, patients who classified as NACT response were called Pre_R and Trm R when they were before and after neoadjuvant chemotherapy; patients who classified as NACT nonresponse were called Pre_SD and Trm_SD when they were before and after neoadjuvant chemotherapy. The clinical characteristics of the patients were summarized in **Table 1**.

**Table 1:** Clinical characteristics of the patients with NACT response and nonresponse.

Clinical characteristics	Patients (N=10)	
	NACT response (n=6)	NACT nonresponse (n=4)
Age (y)	55.5 (46-68)	51 (35-63)
Histopathology		
Squamous carcinoma	5	4
Adenocarcinoma	1	0
Tumor size (cm)	4.45 (3.0-6.9)	6.55 (6.1-9.1)
FIGO stage (2018)		
IIB	1	0
IIIA	1	0
IIIC1	3	4
IIIC2	1	0
Lymphatic metastasis		
Positive	4	4
Negative	2	0
Therapeutic evaluation (CR/PR/SD/PD)	2/4/0/0	0/0/4/0

CR, complete response; PR, partial response; SD, stable disease; PR, progressive disease.

### 2.4 Preparation of serum peptides

Human serum was diluted with 25 mM ABC. DTT was added to a final concentration of 10 mM, and the mixture was heated at 56°C. IAA was added to a final concentration of 20 mM to alkylate free cysteines in the dark for 30 min. The protein mixture was digested by trypsin with an enzyme to protein ratio of 1:50 (w/w) at 37°C overnight. To quench the reaction, 10% (vol/vol) trifluoroacetic acid (TFA) was added to a final concentration of 0.1% followed by centrifugation at 14,000×g for 20 min. C18 Sep-Pak cartridges were then used to desalt the digested products. Subsequently, the peptides were dried by vacuum centrifugation for further enrichment.

### 2.5 Enrichment of N-glycopeptides

For glycopeptide enrichment, the ZIC-HILIC column was equilibrated with 80% ACN containing 1% TFA three times, and glycopeptides were then loaded into the column three times and washed three times with 80% ACN containing 1% TFA. Enriched N-glycopeptides were eluted three times with 0.1% TFA. Subsequently, the enriched N-glycopeptides were dried by vacuum centrifugation and stored at −80°C for further analysis.

### 2.6 LC-ESI-MS/MS analysis

Peptides were resuspended in 0.1% FA and subjected to LC-MS/MS analysis using an Orbitrap Exploris480 mass spectrometer (Thermo Fisher Scientific, MA, USA) coupled to an EASY-NanoLC 1200 system (Thermo Fisher Scientific, MA, USA) with a 75 μm × 50-cm long column (2 μm id). For serum N-glycopeptides, the flow rate was 200 nL/min, and the gradient was 120 min in total (0-90 min, 2%-25% B; 90-115 min, 25-45% B; 115-116 min, 45%-95% B; and 116-126:50 min, 95% B). The MS parameters for N-glycopeptide analysis were set as follows: Orbitrap resolution = 60 k; scan range (m/z) = 400-2000; maximum injection time = 50 ms; normalized AGC target = 300,000; dynamic exclusion after n times, n = 1; dynamic exclusion duration = 15 s. The MS/MS parameters for N-glycopeptide analysis were set as follows: isolation window = 2.2; detector type = Orbitrap; resolution = 15 k; AGC target = 200,000; HCD collision energy = 30% (NCE); stepped collision mode on; and energy difference of ±10%.

### 2.7 Data analysis

Raw data files were searched using Byonic™ software (Protein Metrics, San Carlos, CA, USA), and the mass tolerance of precursor ions and fragment ions was set as 10 ppm and 20 ppm, respectively. Trypsin was selected as the enzyme with a maximum of two missed cleavages allowed. The fixed modification was carbamidomethyl (C), and the variable modifications included oxidation (M), deamidation (N, Q) and N-glycan modifications (N). A human N-glycan database (from the Byonic database) containing 57 human plasma N-glycans was employed. The results were filtered at a confidence threshold of Byonic score >150, Delta modification score >10, PEP2D < 0.05 and FDR2D < 0.01 in Byologic (Protein Metrics) software, which inputs both MS1 raw data and Byonic search results. The theoretical m/z of the oxonium ions from GlcNAc (m/z 138.05, m/z 168.05 and m/z 204.09), NeuAc (m/z 274.09 and 292.10), GlcNAc-Hex (m/z 366.14), HexHexNAcFuc (m/z 512.20) and HexNAcHexNeuAc (m/z 657.23) in glycopeptides from HCD-MS are known for glycan identification.

## 3 Results

### 3.1 Framework for intact glycopeptide analysis

**Figure 1:**
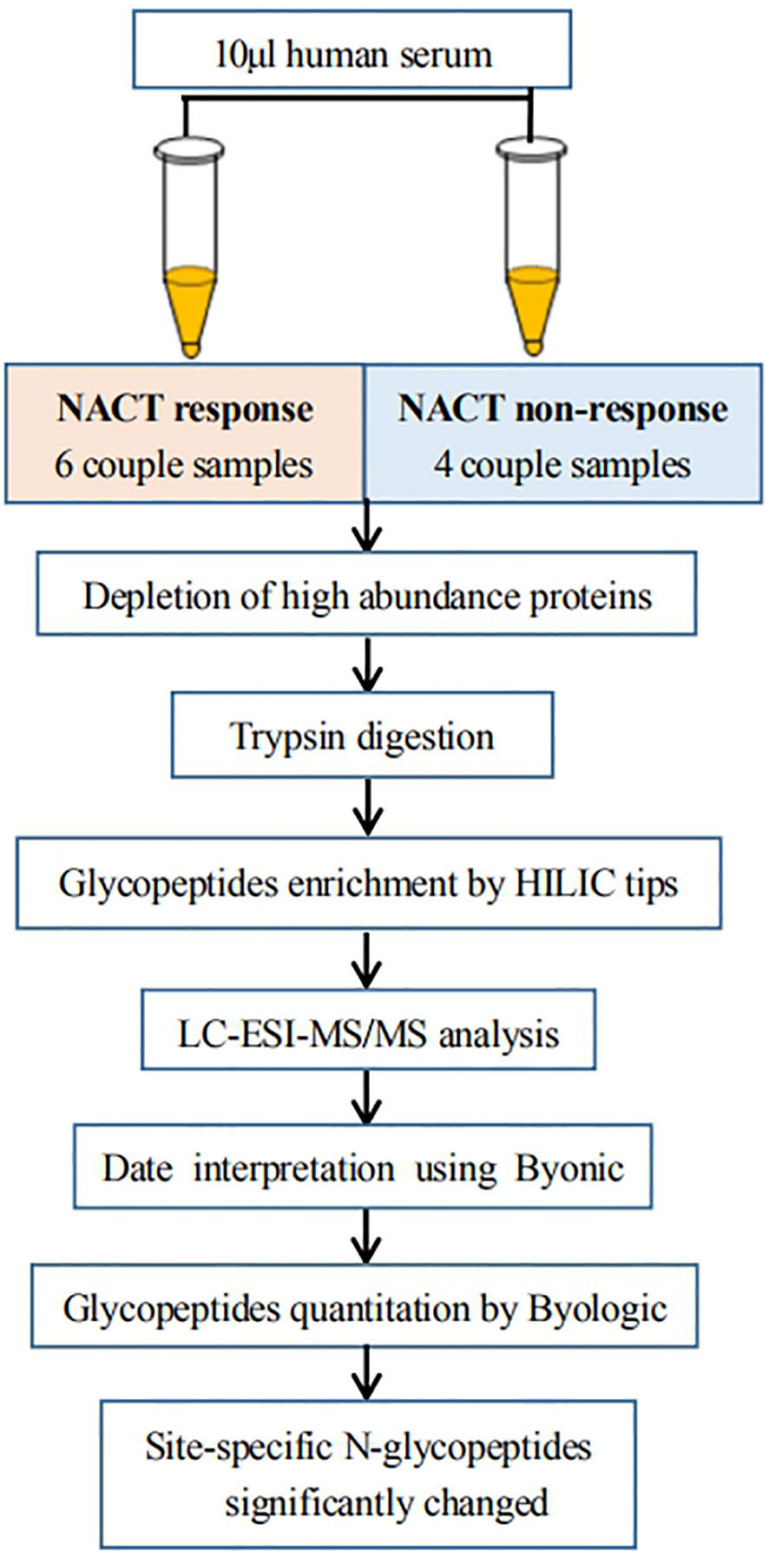
Workflow of quantitative LC-ESI-MS/MS analysis of intact N-glycopeptide derived from the sera between NACT response and NACT nonresponse patients.

To reduce the sample preparation steps and improve the accuracy of quantification, as shown in **Figure 1**, we developed a workflow for large-scale intact N-glycopeptide identification, which included high-abundance protein depletion, glycopeptide enrichment, offline peptide fractionation and LC-ESI-MS/MS for differential determination of glycosylation composition changes in LACC patient sera between NACT responsive and nonresponsive patients. Ten pairs of serum samples from 10 patients before and after NACT were included. To further investigate these glycopeptides, we performed a quantitative analysis. The detection of low-abundance glycopeptides in complex mixtures is difficult due to the suppression of glycopeptide mass spectral signals in the presence of nonglycopeptides. To overcome this issue, highly efficient depletion of high-abundance proteins and enrichment of glycopeptides are needed.

### 3.2 Glycopeptide distribution based on the byonic score

**Figure 2:**
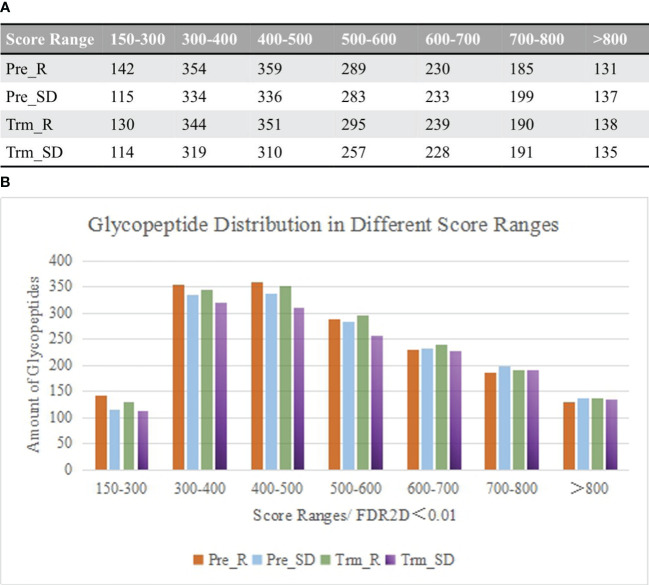
**(A)** Comparison of the distribution of glycopeptides in different score ranges among the samples between chemotherapy responsive and nonresponsive ones before and after NACT. **(B)** Column diagram shows the top 2 number of glycopeptides that are distributed between score 300-500.

Raw spectra of complex samples obtained from the DDA mode were retrieved by Byonic software. The results were filtered at a confidence threshold of Byonic score >150, Delta modification score >10, PEP2D < 0.05 and FDR2D < 0.01. Base on retention time, the distribution of glycopeptide scores is shown in **Figure 2A**.

As the patient serum contains a large number of glycopeptides, the Byonic score was set greater than 150 to increase the credibility of the results. As shown in **Figure 2B**, the fractions ranging from 300 to 500 contained the largest number of glycopeptides. Under these conditions, data on the distribution of glycosylation types were also obtained, including the number of glycoproteins, glycosites, glycopeptides, percentage of fucosylated glycopeptides and percentage of sialylated glycopeptides.

### 3.3 Glyco-distribution in human serum from samples

**Figure 3:**
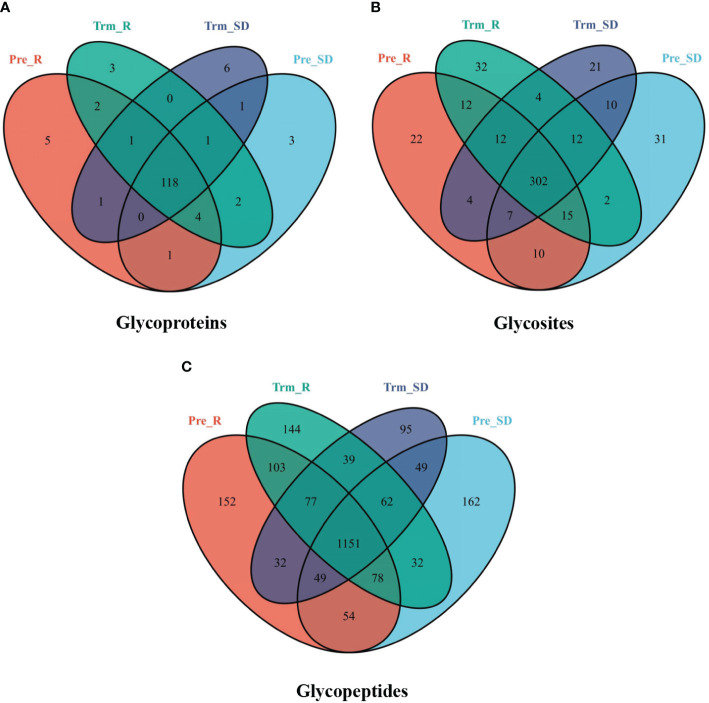
Venn diagram showing the number of **(A)** glycoproteins, **(B)** glycosites, and **(C)** glycopeptides from the NACT response (red), NACT nonresponse (blue) before treatment and NACT response (green), NACT nonresponse (purple) after treatment samples.

In the present study, we first performed a qualitative analysis. The component Venn diagram of the NACT responsive and nonresponsive groups before and after NACT is shown in **Figure 3**. To overcome the inhibition of signals by nonglycopeptides to facilitate glycopeptide detection, we combined ZIC-HILIC-based glycopeptide extraction to improve the MS signal of the glycopeptides. HILIC is a well-recognized technique for effectively enriching glycans and glycopeptides (24). Because many of these glycopeptides were in low abundance, we used this method to enrich the glycopeptides to enhance the mass spectrometry signal.

In total, 148 glycoproteins were identified, including 138 in the NACT responsive group and 138 in the NACT nonresponsive group. There were 13 differentially expressed glycoproteins in the NACT responsive group and 18 in the NACT nonresponsive group before and after chemotherapy (**Figure 3A**). A total of 2,279 glycopeptides were identified, including 1,973 in the NACT responsive group and 1,880 in the NACT nonresponsive group. The number of differentially expressed glycopeptides before and after chemotherapy was 654 in the NACT-responsive group and 569 in the NACT-nonresponsive group (**Figure 3C**). These identifications also corresponded to 496 glycosites, including 434 in the NACT responsive group and 430 in the NACT nonresponsive group. The differential glycosites before and after chemotherapy were 93 in the NACT responsive group and 99 in the NACT nonresponsive group (**Figure 3B**).

### 3.4 Glycopeptides identified as potential biomarker candidates by LC-ESI-MS/MS

To discover the differential glycopeptides for therapeutic predictive markers between NACT responsive and nonresponsive patients, LC-ESI-MS/MS was performed for quantitative analysis among individual patients. In a total of 10 pairs of serum samples (including 2 CR, 4 PR and 4 SD), we analyzed the results of quantified glycopeptides by Byologic software. These patient samples were classified into four types as follows: NACT response before treatment (n=6), NACT response after treatment (n=6), NACT nonresponse before treatment (n=4) and NACT nonresponse after treatment (n=4). The relative abundance of the glycopeptides was compared by *t test*.

In the present study, 20 glycopeptides had a significant difference between the NACT responsive and nonresponsive groups before treatment according to the LC-ESI-MS/MS results (**Table 2**). In total, 26 glycopeptides had a significant difference before and after treatment in the NACT responsive group (**Table 3**), and 10 glycopeptides had a significant difference before and after treatment in the NACT nonresponsive group (**Table 4**). After comparison, six differential glycopeptides (MASP1, LUM, ATRN, CO8A, CO8B and CO6) were screened out because they were differential in NACT responsive groups before and after treatment as well as in two groups before NACT (**Table 5**). There was no overlap with the glycopeptides that differed before and after chemotherapy in the NACT nonresponsive group.

**Table 2:** List of the difference glycopeptides between NACT responsive and nonresponsive group before chemotherapy.

No	Protein name	Sequence	Glycans	*P* value
1	ATRN_HUMAN	K.IDSTGnVTNELR.V	HexNAc(2)Hex(5)	0.021
2	IGJ_HUMAN	R.EnISDPTSPLR.T	HexNAc(4)Hex(5)Fuc(1)NeuAc(2)	0.017
3	A1AG1_HUMAN	R.NEEYnK.S	HexNAc(4)Hex(5)NeuAc(1)	0.045
4	A1AG1_HUMAN	R.NEEYnK.S	HexNAc(4)Hex(5)NeuAc(2)	0.010
5	C4BPA_HUMAN	R.FSLLGHASISCTVEnETIGVWR.P	HexNAc(4)Hex(5)NeuAc(2)	0.048
6	PROC_HUMAN	K.EVFVHPnYSK.S	HexNAc(4)Hex(5)NeuAc(2)	0.021
7	CO8A_HUMAN	R.GGSSGWSGGLAQnR.S	HexNAc(4)Hex(5)NeuAc(1)	0.019
8	CO8B_HUMAN	R.nVTEK.M	HexNAc(4)Hex(5)NeuAc(1)	0.018
9	CO6_HUMAN	K.VLnFTTK.A	HexNAc(4)Hex(5)NeuAc(1)	0.035
10	CO6_HUMAN	R.LSSnSTK.K	HexNAc(4)Hex(5)NeuAc(2)	0.015
11	PZP_HUMAN	K.VnITVCGEYTYGK.P	HexNAc(3)Hex(5)NeuAc(1)	0.014
12	C4BPB_HUMAN	K.TLFCnASK.E	HexNAc(4)Hex(5)NeuAc(2)	0.015
13	C4BPB_HUMAN	R.LGHCPDPVLVNGEFSSSGPVnVSDK.I	HexNAc(4)Hex(5)NeuAc(2)	0.001
14	CPN2_HUMAN	R.AFGSNPnLTK.V	HexNAc(4)Hex(5)NeuAc(1)	0.007
15	PEDF_HUMAN	K.VTQnLTLIEESLTSEFIHDIDR.E	HexNAc(4)Hex(5)NeuAc(2)	0.014
16	MASP1_HUMAN	R.NnLTTYK.S	HexNAc(4)Hex(5)NeuAc(2)	0.001
17	LUM_HUMAN	K.LHINHNnLTESVGPLPK.S	HexNAc(4)Hex(5)Fuc(1)NeuAc(1)	0.010
18	PHLD_HUMAN	K.LNVEAAnWTVR.G	HexNAc(4)Hex(5)NeuAc(1)	0.013
19	LG3BP_HUMAN	K.AAIPSALDTnSSK.S	HexNAc(5)Hex(6)NeuAc(2)	0.030
20	FCGBP_HUMAN	R.VITVQVAnFTLR.L	HexNAc(4)Hex(5)Fuc(1)NeuAc(1)	0.006

**Table 3:** List of the difference glycopeptides before and after chemotherapy in NACT responsive group.

No	Protein name	Sequence	Glycans	*P* value
1	ATRN_HUMAN	K.CEnLTTGK.H	HexNAc(4)Hex(5)NeuAc(1)	0.047
2	ATRN_HUMAN	K.IDSTGnVTNELR.V	HexNAc(2)Hex(5)	0.041
3	IGG1_HUMAN	R.EEQYnSTYR.V	HexNAc(4)Hex(4)Fuc(1)	0.022
4	C1R_HUMAN	K.EHEAQSnASLDVFLGHTNVEELMK.L	HexNAc(4)Hex(5)NeuAc(2)	0.044
5	C1R_HUMAN	R.CnYSIR.V	HexNAc(4)Hex(5)NeuAc(1)	0.030
6	CFAB_HUMAN	R.SPYYnVSDEISFHCYDGYTLR.G	HexNAc(4)Hex(5)NeuAc(1)	0.027
7	IGHA1_HUMAN	R.PALEDLLLGSEAnLTCTLTGLR.D	HexNAc(5)Hex(5)NeuAc(1)	0.045
8	C1QA_HUMAN	R.RNPPMGGNVVIFDTVITNQEEPYQnHSGR.F	HexNAc(4)Hex(5)Fuc(1)NeuAc(1)	0.028
9	FETUA_HUMAN	R.KVCQDCPLLAPLnDTR.V	HexNAc(4)Hex(5)NeuAc(2)	0.048
10	A1BG_HUMAN	R.EGDHEFLEVPEAQEDVEATFPVHQPGnYSCSYR.T	HexNAc(4)Hex(5)NeuAc(1)	0.024
11	CFAI_HUMAN	K.LISnCSK.F	HexNAc(4)Hex(5)NeuAc(2)	0.043
12	CFAI_HUMAN	K.LSDLSInSTECLHVHCR.G	HexNAc(4)Hex(5)NeuAc(2)	0.020
13	THBG_HUMAN	K.VTACHSSQPnATLYK.M	HexNAc(4)Hex(5)NeuAc(1)	0.005
14	CO2_HUMAN	K.QSVPAHFVALnGSK.L	HexNAc(2)Hex(5)	0.043
15	CO8A_HUMAN	R.GGSSGWSGGLAQnR.S	HexNAc(4)Hex(5)NeuAc(1)	0.001
16	CO8B_HUMAN	R.nVTEK.M	HexNAc(4)Hex(5)NeuAc(1)	0.019
17	CFAH_HUMAN	K.IPCSQPPQIEHGTInSSR.S	HexNAc(4)Hex(5)NeuAc(2)	0.022
18	CO6_HUMAN	K.VLnFTTK.A	HexNAc(4)Hex(5)NeuAc(1)	0.026
19	CO6_HUMAN	R.LSSnSTK.K	HexNAc(4)Hex(5)NeuAc(2)	0.028
20	HGFL_HUMAN	R.AFHYnVSSHGCQLLPWTQHSPHTR.L	HexNAc(4)Hex(5)NeuAc(2)	0.010
21	PROP_HUMAN	K.YPPTVSMVEGQGEKnVTFWGR.P	HexNAc(4)Hex(5)Fuc(1)NeuAc(1)	0.047
22	MASP1_HUMAN	R.NnLTTYK.S	HexNAc(4)Hex(5)NeuAc(2)	0.014
23	LUM_HUMAN	K.KLHINHNnLTESVGPLPK.S	HexNAc(4)Hex(5)Fuc(1)NeuAc(1)	0.000
24	LUM_HUMAN	K.KLHINHNnLTESVGPLPK.S	HexNAc(4)Hex(5)Fuc(1)NeuAc(2)	0.010
25	LUM_HUMAN	K.LHINHNnLTESVGPLPK.S	HexNAc(4)Hex(5)Fuc(1)NeuAc(1)	0.005
26	LG3BP_HUMAN	K.AAIPSALDTnSSK.S	HexNAc(4)Hex(5)Fuc(1)NeuAc(1)	0.011

**Table 4:** List of the difference glycopeptides before and after chemotherapy in NACT nonresponsive group.

No	Protein name	Sequence	Glycans	*P* value
1	IGG1_HUMAN	R.EEQYnSTYR.V	HexNAc(4)Hex(4)Fuc(1)	0.031
2	CO3_HUMAN	K.TVLTPATNHMGnVTFTIPANR.E	HexNAc(2)Hex(5)	0.029
3	IGHM_HUMAN	K.YKnNSDISSTR.G	HexNAc(4)Hex(5)Fuc(1)NeuAc(1)	0.033
4	FINC_HUMAN	R.DQCivDDITYNVnDTFHK.R	HexNAc(4)Hex(5)NeuAc(1)	0.046
5	CFAI_HUMAN	K.LISnCSK.F	HexNAc(4)Hex(5)NeuAc(2)	0.003
6	CO2_HUMAN	K.QSVPAHFVALnGSK.L	HexNAc(2)Hex(5)	0.039
7	CBG_HUMAN	R.AQLLQGLGFnLTER.S	HexNAc(4)Hex(5)NeuAc(1)	0.045
8	C4BPB_HUMAN	R.LGHCPDPVLVNGEFSSSGPVnVSDK.I	HexNAc(4)Hex(5)NeuAc(2)	0.027
9	ZA2G_HUMAN	R.FGCEIENnR.S	HexNAc(4)Hex(5)NeuAc(2)	0.046
10	ITIH3_HUMAN	K.TAFITnFTLTIDGVTYPGNVK.E	HexNAc(4)Hex(5)NeuAc(2)	0.032

**Table 5:** List of the glycopeptides be different before and after chemotherapy in NACT responsive group, as well as in two groups before NACT.

No	Protein name	Sequence	Glycans
1	ATRN_HUMAN	K.IDSTGnVTNELR.V	HexNAc(2)Hex(5)
2	CO8A_HUMAN	R.GGSSGWSGGLAQnR.S	HexNAc(4)Hex(5)NeuAc(1)
3	CO8B_HUMAN	R.nVTEK.M	HexNAc(4)Hex(5)NeuAc(1)
4	CO6_HUMAN	R.LSSnSTK.K	HexNAc(4)Hex(5)NeuAc(2)
5	MASP1_HUMAN	R.NnLTTYK.S	HexNAc(4)Hex(5)NeuAc(2)
6	LUM_HUMAN	K.LHINHNnLTESVGPLPK.S	HexNAc(4)Hex(5)Fuc(1)NeuAc(1)

**Figure 4:**
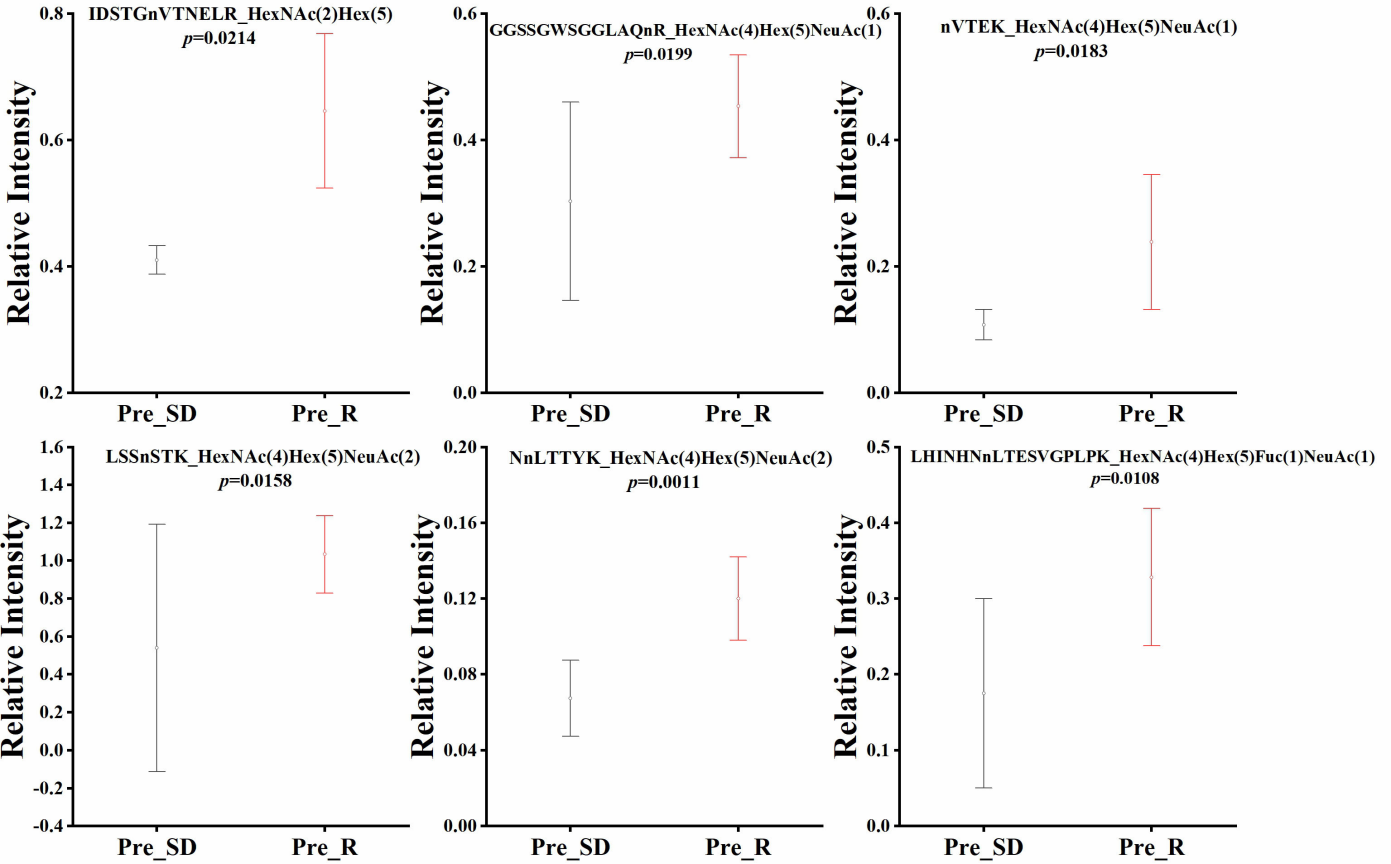
Box plot of relative abundance of N-glycopeptide in serum between NACT responsive (Pre_R) and nonresponsive (Pre_SD) patients before chemotherapy.

**Figure 5:**
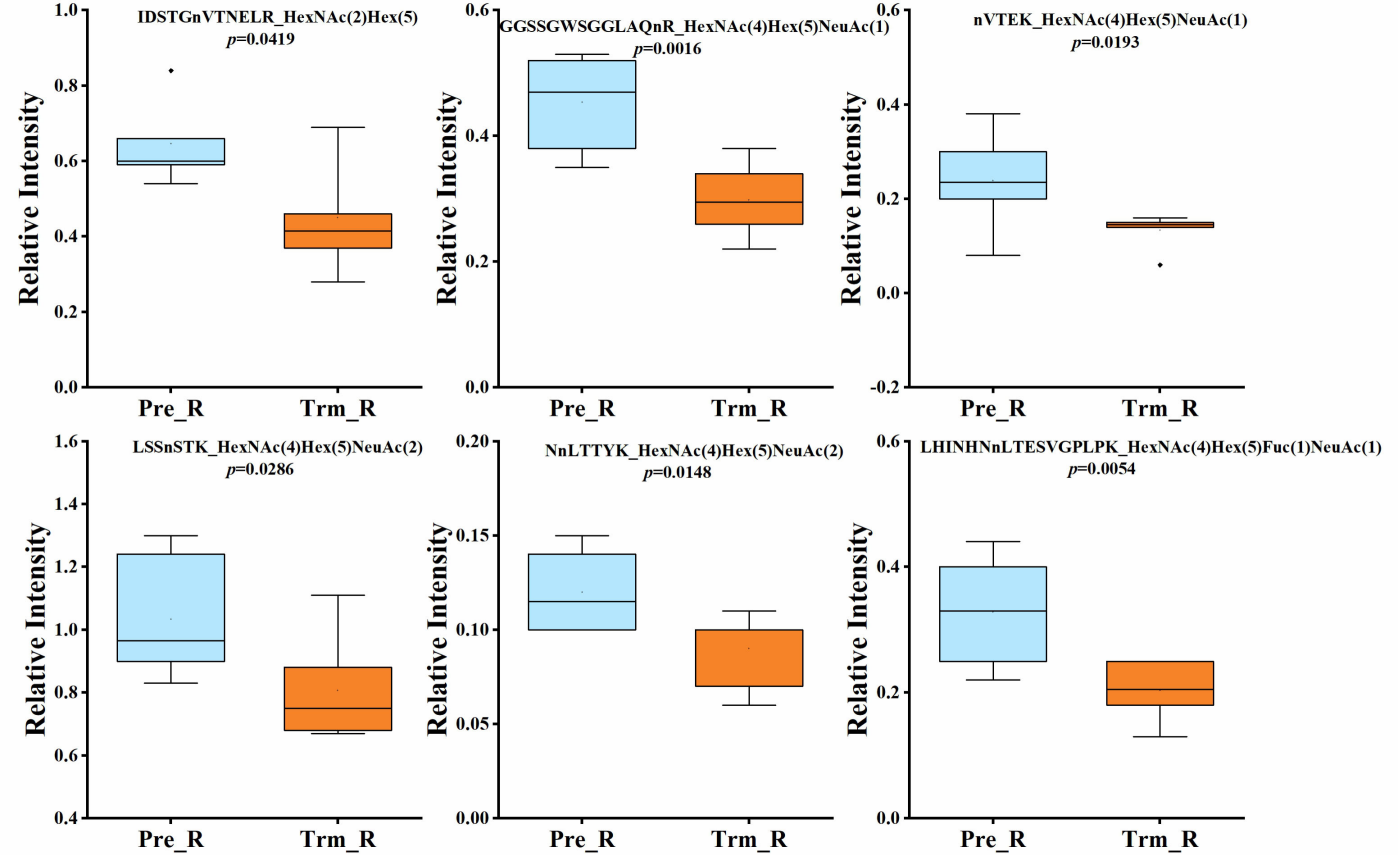
Box plot of relative abundance of N-glycopeptide in serum of NACT responsive patients before (Pre_R) and after chemotherapy (Trm_R).

Quantitative statistical analysis (*P values* were obtained by *t test*) was performed on the relative abundance of these six glycopeptides, and we found that the relative abundance of the six glycopeptides was significantly higher in the NACT responsive group than in the NACT nonresponsive group before treatment (**Figure 4**). In the NACT responsive group, the relative abundance of all six glycopeptides was significantly decreased after chemotherapy (**Figure 5**).

### 3.5 Distribution of glycosylation types in the human serum samples

**Figure 6:**
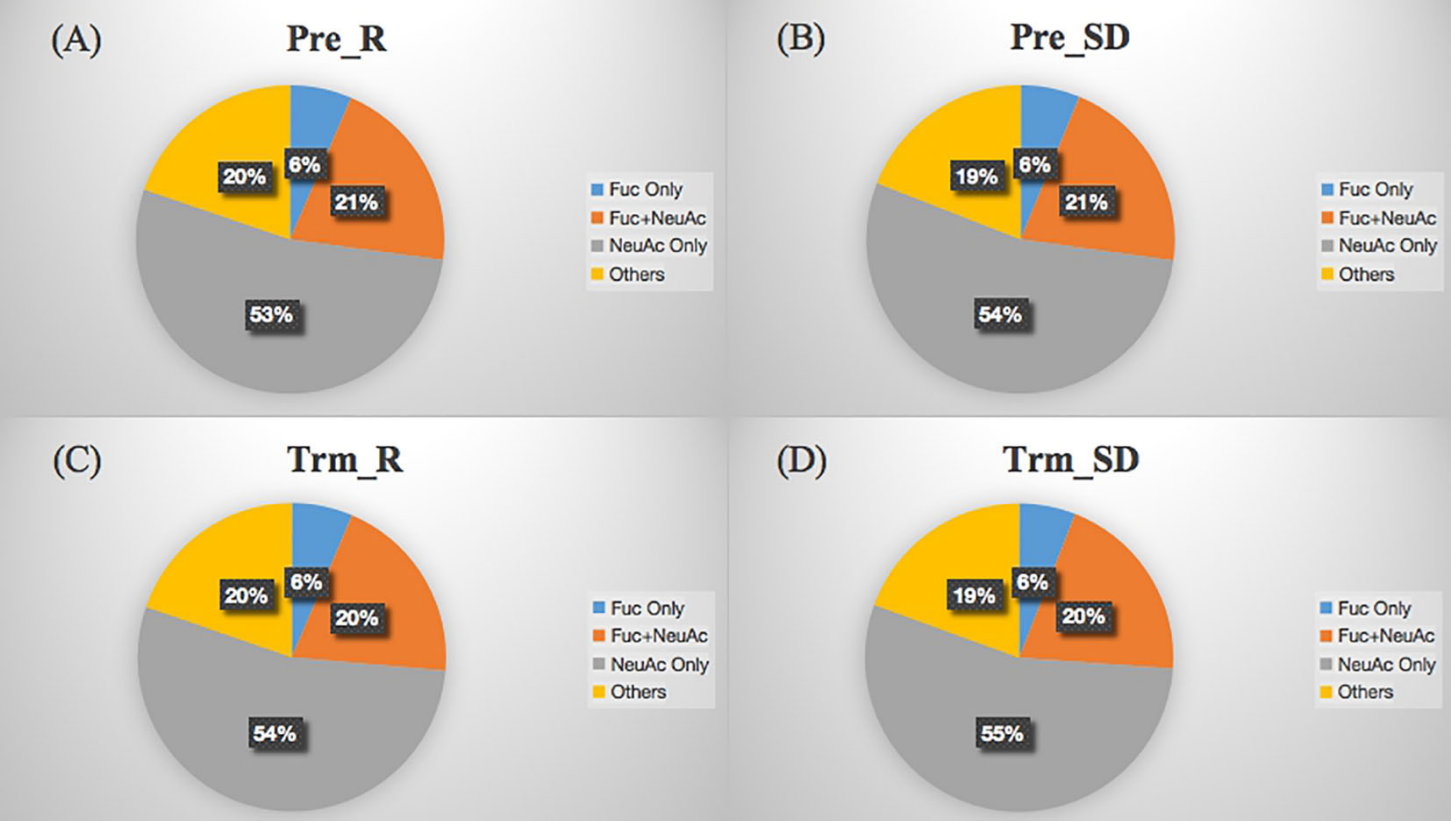
Distribution of glycosylation types of sera glycoproteins from NACT response (**A**, Pre_R), NACT nonresponse (**B**, Pre_SD) before treatment and NACT response (**C**, Trm_R), NACT nonresponse (**D**, Trm_SD) after treatment. The labels Fuc only, Fuc+NeuAc, NeuAc only and Others refer to the glycopeptides fucosylated only, fucosylated and sialylated, sialylated only and other glycosylated types, respectively.

The site-specific glycosylation distribution of serum glycoproteins involves the number of glycopeptides, glycoproteins, glycosylation types and the proportion of glycosylated or sialylated glycopeptides. As shown in **Figure 6**, NeuAc was the dominant glycosylation type in LACC patient sera, and there was no significant difference in glycosylation sites and glycopeptide amounts of glycoproteins before and after NACT in the NACT response and NACT nonresponsive groups.

## 4 Discussion

Some studies have reported that LACC patients who respond to NACT benefit from NACT with improved OS and PFS compared to nonresponders. As a result, it is essential to identify which LACC patients may respond to chemotherapy and investigate common biomarkers to distinguish NACT responders.

In the present study, 26 glycopeptides were significantly different before and after treatment in the NACT-responsive group. Among these, six glycopeptides were screened out because they were differentially expressed not only in the NACT-responsive groups before and after treatment but also in the NACT responder and nonresponder groups before treatment. Thus, MASP-1, LUM, ATRN, CO8A, CO8B and CO6 may be used as promising glycopeptide biomarkers to distinguish NACT-responsive LACC patients who may benefit from NACT followed by CCRT. The relative abundances of the six glycopeptides, including MASP1, LUM, ATRN, CO8A, CO8B and CO6, were significantly higher in the NACT-responsive group and were significantly decreased after chemotherapy. High levels of these six glycopeptides may indicate that chemotherapy is effective. The innovation of the present study was the identification of soluble glycopeptides for LACC, which can be used to screen the efficacy of chemotherapy. Abnormal glycosylation is associated with tumor malignancy and disease progression. Glycosylation is one of the most extensive and important posttranslational modifications of proteins in organisms, accounting for 70% in the human body (25). Most glycans are abundant on the surface and cytoplasm of the cells. Glycan regulation has been confirmed to participate in various physiological processes. Glycans are composed of the galactose (Gal), N-acetylgalactosamine (GalNAc), glucose (Glc), fucose (Fuc) and mannose (Man) monosaccharides and their derivatives, including glucuronic acid (GlcA), xylose and sialic acids. The main classes of glycoproteins consist of at least five major types, namely, N-glycans, O-glycans, glycosaminoglycan, GPI-anchored proteins and O-linked GlcNAcylation (26). Site-specific glycosylation changes may become a promising predictive biomarker in clinical diagnosis and treatment. For instance, several studies have revealed site-specific glycosylation structural changes in serum glycoproteins as potential markers for early HCC, including ceruloplasmin, kininogen-1, α-1-antitrypsin and vitronectin (27). Recently, advances in mass spectrometry have enabled the discovery of site-specific glycopeptide markers for early cancer detection. The fucosylated and sialylated glycan structures of serum Hp have been shown to be significantly elevated in patients with HCC compared to patients with cirrhosis (28, 29).

In cervical cancer, glycosylation is also involved in cell metabolism, signal transduction, tumor cell division, tumor cell metastasis, immune regulation, cancer diagnosis and cancer management (30). It has been reported that the α2,6-terminal sialylation and fucosylation patterns of intracellular proteins exist in cervical cancer tissue rather than normal cervix tissue (31). In addition, elevated OGT and O-GlcNAcylation are associated with increased cell proliferation and reduced cellular senescence in HPV-induced cervical cancer (32). Glycosylation in cervical cancer also has significant implications for cancer treatment. For example, galectin-7 produces a CCRT response and acts as a significant predictor for cervical cancer patients treated with definitive radiation therapy (33).

Therefore, the present study aimed to investigate the glycosylation difference in the serum of NACT responders and nonresponders. In addition, we investigated potential glycopeptide biomarkers to distinguish LACC patients who will benefit from NACT followed by CCRT.

In the present study, we analyzed the site-specific N-glycosylation of serum in patients with locally advanced cervical cancer before and after chemotherapy. It was found that more than half of the glycosites were sialic acid followed by fucose. The relative abundance of glycopeptides was significantly higher in the NACT-responsive group before chemotherapy than in the NACT-nonresponsive group. Six potential glycopeptide biomarkers were quantified both in the chemosensitive group before and after chemotherapy as well as in the two groups before chemotherapy. The corresponding proteins included MASP-1, LUM, ATRN, CO8A, CO8B and CO6. Five of the six glycosites in these six differential glycopeptides contained sialic acid, and one glycosite contained fucose. The most significant cancer-associated changes in glycosylation are fucosylation, galactosylation, and sialylation (34). It is well known that sialic acid plays an important role in immune escape, virus infection, tumorigenesis and development (35). Also, compared with IgG glycans of non-response group, agalactosylated N-glycans increased while monosialylated N-glycans and digalactosylated N-glycans decreased in the response group of local advanced gastric cancer (LAGC) patients (34). According to a previous report, fucose is closely related to the proliferation, metastasis and immune response of tumor cells (36). The abnormal expression of glycosyltransferases (GTs) leading to aberrant glycosylation patterns are considered a marker of cancer. Recently, studies have found that GTs are involved in mediating multidrug resistance (MDR) in cancer cells through complex mechanisms and can influence therapeutic effect (37). Schultz et al. reported that ST6Gal I overexpression is a hallmark of ovarian cancer, and it is closely related to cisplatin-induced cell death. The overexpression of ST6Gal I reduced the activation of caspase 3, and it protected against cell death after cisplatin treatment. This indicates that ST6Gal I may be a novel contributor to cisplatin resistance (38). Aloia et al. reported that increased expression of ST3Gal II in breast cancer could be predictive markers of poor prognosis (39). However, the biological mechanism for such a relationship between glycosylation and cancers has yet to be determined. In the future, we will strengthen cooperation with experts in the field of tumor resistance, and hope that we can further elucidate the mechanism.

The biological function of the six differential glycopeptides detected in the present study plays a key role in tumor occurrence and progression. Patients with higher serum MASP-1 levels may have exacerbated complement activation, which leads to basement membrane disorder and eventually to basement membrane injury or rupture, resulting in tumor invasion. A previous study has reported that the expression of MASP-1 is significantly increased in cervical cancer patients, HPV-positive patients and cervical secretory tissues with late FIGO stage (stage III - IV) (40). Similarly, the expression of MASP-1 in invasive cervical cancer is significantly higher than that in precancerous lesions, and higher serum levels of MASP-1 are associated with poor prognosis in cervical cancer (41). In the present study, the serum levels of MASP-1-related glycopeptides in the NACT-responsive group decreased significantly after chemotherapy, which was consistent with previous reports. The present results further illustrated the differences in glycopeptide sequences and the corresponding glycans. Recently, increasing experimental data have shown that LUM is expressed in various types of tumor tissues. LUM not only regulates extracellular water balance and collagen fiber formation but also impacts tumor growth, adhesion and migration (42). We found that after NACT, the relative abundance of LUM-related glycopeptides in the NACT-responsive group significantly decreased, indicating that the prognosis of LACC may be improved by inhibiting LUM-related glycopeptides. ATRN is involved in innate immune cell aggregation in the inflammatory response and may regulate the chemotactic activity of chemokines (43). ATRN is a predictive biomarker in prostate cancer because it distinguishes prostate cancer from benign prostatic hyperplasia (44). Complement C8 (CO8A and CO8B) and CO6 are members of the complement gene family; they are the main components of the membrane attack complex, and they are also part of the innate immune system. The complement of innate immunity exists in the tumor microenvironment, and the function of complement depends on the type of tumor and its heterogeneity, which may be antitumor or protumor (45). C8A is also used as a biomarker for predicting the benefit of trastuzumab in HER2-positive breast cancer patients (46). High C8A expression has a significant benefit in disease-free survival. Studies have shown that the expression level of C8B has a protective effect on the overall survival and recurrence-free survival of patients with HBV-related hepatocellular carcinoma (47). In the present research, we further illustrated the differences in glycopeptide sequences, including K.IDSTGnVTNELR.V, R.GGSSGWSGGLAQnR.S, R.nVTEK.M, R.LSSnSTK.K, R.NnLTTYK.S, K.LHINHNnLTESVGPLPK.S and the corresponding glycans. To our knowledge, this is the first study to demonstrate that serum intact N-glycosylation may be correlated with response to NACT in LACC. Some of these altered N-glycopeptides have the potential to predict efficacy of NACT.

The present study highlighted the application of LC-MS/MS for the discovery of N-glycopeptides associated with LACC. After quantitative analysis, 6 of 570 glycopeptides were identified as biomarkers for predicting the sensitivity of neoadjuvant chemotherapy in LACC. The relative abundance of the six glycopeptides (corresponding proteins including MASP1, LUM, ATRN, CO8A, CO8B and CO6) was significantly higher in the NACT responsive group and was significantly decreased after chemotherapy. High levels of these six glycopeptides may indicate that chemotherapy is effective. In future studies, we will verify these glycopeptides by parallel reaction monitoring (RPM) to further improve the diagnostic efficacy. Additional biological mechanism studies should be performed to investigate how glycopeptides play a role in NACT responders.

## Data availability statement

The original contributions presented in the study are included in the article/supplementary material. Further inquiries can be directed to the corresponding authors.

## Ethics statement

The study was approved by Shanghai Jiaotong University School of Medicine affiliated with the Ruijin Hospital Ethics Committee (N-2018-239). The patients/participants provided their written informed consent to participate in this study.

## Author contributions

WF and HJL contributed to conception and design of the study. JL and XF organized the database and performed the statistical analysis. WF and HL assisted with provision and supervision of the study patients. CZ stored and processed the specimens. JL and XF wrote the first draft of the manuscript. WF, HJL, and YJ wrote sections of the manuscript. All authors contributed to manuscript revision, read, and approved the submitted version.
